# 4-Aminopyridine improves real-life gait performance in SCA27B on a single-subject level: a prospective n-of-1 treatment experience

**DOI:** 10.1007/s00415-023-11868-y

**Published:** 2023-07-13

**Authors:** Jens Seemann, Andreas Traschütz, Winfried Ilg, Matthis Synofzik

**Affiliations:** 1 Section Computational Sensomotorics, Hertie Institute for Clinical Brain Research, Otfried-Müller-Straße 25, 72076 Tübingen, Germany; 2 Centre for Integrative Neuroscience (CIN), Tübingen, Germany; 3 Division Translational Genomics of Neurodegenerative Diseases, Hertie-Institute for Clinical Brain Research and Center of Neurology, University of Tübingen, Tübingen, Germany; 4 German Center for Neurodegenerative Diseases (DZNE), Tübingen, Germany

Dear Sirs,

While effective treatments are still rare in degenerative ataxias, the identification of the underlying gene defect in an increasing number of ataxias [1] allows for targeted treatment developments and evaluations as part of a “precision medicine for ataxia” [2]. In particular, the recently identified SCA27B, caused by GAA-repeat expansions in *FGF14* [3, 4], might not only represent one of the most common genetic causes of ataxia—even in late-onset, seemingly sporadic ataxia patients—[2, 3], but also respond at least partially to 4-aminopyridine (4-AP) [2]. Previous work has shown that 4-AP might be effective in several neurological disorders, including certain types of cerebellar ataxia (review in [5]), with genetic stratification (e.g., for GAA-*FGF14* repeat expansions) now even potentially allowing to predict which ataxia patient might respond to 4-AP.

However, objective quantitative evidence for the efficacy of 4-AP in SCA27B—as well as in other ataxias—is still starkly absent, inter alia due to the scarcity of pertinent outcome measures. For evaluating the treatment effects in both clinical trials and individual patient treatment settings, sensitive outcome measures have to be identified that show a change meaningful to patients [6]. Gait assessment and in particular analysis of real-life walking behavior—which is ecologically more meaningful than in-clinic or lab assessments—can provide meaningful outcome measures for evaluating treatment interventions, as cerebellar ataxia patients report gait and functional mobility impairments as having the greatest impact on their daily lives [7–9]. In line with this notion, gait and balance impairments also contribute the by far most frequent impairment in SCA27B, reported in 44/46 SCA27B patients in a recent study [2].

Here, we present prospective single-subject gait sensor data in different walking conditions (including patient’s everyday life) with vs without 4-AP, demonstrating that (i) 4-AP improves ataxia-related gait characteristics in SCA27B, and that (ii) gait parameters assessable by body-worn sensors allow to capture the 4-AP on-/off effects also in—ecologically highly relevant—real-life conditions, even on a single-subject level.

Assessments of this single-subject (= n-of-1) treatment were applied between June 2021 and April 2023 at the ataxia outpatient clinics, Center of Neurology, University Tübingen, as an “individual trial of therapy” under a structured named patient treatment protocol (German: individueller Heilversuch), which can be methodologically characterized as a prospective open-label single-subject experience [10].

The 63-year-old man presented with a 5-year history of progressive balance problems and horizontal diplopia, both reported by the patient to partly worsen with exercise and exhaustion. These impairments were increasingly accompanied over time by progressive fine motor impairment, dysarthria, and dysphagia. The patient also complained of recurrent feeling of “brain fog”, particularly in the mornings, but did neither report nor show other cognitive dysfunctions. Based on an autosomal-dominant history for ataxia, and non-conclusive results on whole exome and genome sequencing, GAA-repeat expansion testing in *FGF14* was performed according to previously reported standard methods [11], demonstrating a heterozygous repeat expansion of 330 repeats confirming SCA27B. Based on prior mixed experiences with at least some degenerative ataxia patients in our ataxia clinics responding to 4-aminopyridine (4-AP) [12], 4-AP treatment (10 mg extended release, twice daily) was initiated in this patient at age 63 years (July 2021; prior to the genetic diagnosis).

Assessments of the patient, performed in a prospective longitudinal fashion, included three visits: visit #1 prior to taking the drug (June 2021) (“NO” drug); visit #2 on drug (March 2023; drug has been taken for 21 months by then) (“ON” drug); visit #3 when the patient had paused taking the drug for 20 h (April 2023) (“OFF” drug). Assessments included: laboratory-based gait recordings by body-worn sensors (all 3 visits), clinical ataxia severity (Scale for the Assessment and Rating of Ataxia; SARA [13]) (all 3 visits), real-life gait recordings (ON and OFF visits #2 and #3); patient's impression of disease severity (PGI-S) [14] for the global disease status and for ataxia functional domains of key importance for SCA27B patients (speech, upper limb function and gait) (PGI-S domains), each documented on a 5-point Likert scale to rate severity (not affected, mildly affected, moderately affected, severely affected, and very severely affected) [14] (ON and OFF visits #2 and #3).

Gait movements were assessed by three body-worn inertial sensors (APDM’s Opal^®^ V2C System with Mobility Lab, Clario, San Matteo, United States) attached on both feet, and posterior trunk at the level of L5 in three gait conditions: (1) instructed laboratory walking (LBW); (2) supervised free walking (6–8 min) in an institutional (hospital) compound environment (SFW); and (3) real-life walking behavior in patients’ real life during their everyday living (RLW). Detailed description of walking conditions and inertial sensors can be found in [15]. In the LBW condition, we assessed gait in preferred and slow speed, as previous studies demonstrated increased step variability and body sway for slow gait (as opposed to preferred speed) [16, 17]. In real-life walking (RLW), the patient recorded 3–4 h of movements during everyday living, which included for each RLW assessment timepoint the same 30-min walk with over 1600 gait cycles.

To compare the single patient's gait data with other ataxia patients as disease reference cohort, we included in the analysis data from a population with degenerative cerebellar ataxia (ATX, *N* = 43) (cohort data previously published in [15]) for all three walking conditions (see Fig. 1).

**Fig. 1:**
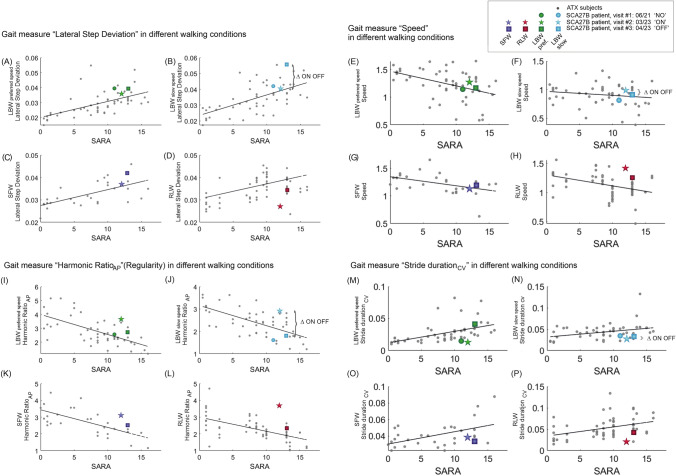
Results of the gait measures *lateral step deviation* (**A**–**D**, higher variability values indicate worse gait performance), *speed* (**E**–**H**), *harmonic ratio*_AP_ (**I**–**L**, higher values indicate increased regularity and better gait performance) and *stride duration*_*CV*_ (**M**–**P**, higher variability values indicate worse gait performance) for the different walking conditions (color coded): laboratory-based walking (LBW) with preferred and slow speed; Supervised free walking (SFW); Real-Life walking (RLW). Gait measures are shown on the *y*-axis over the SARA scores on the *x*-axis. Gait measures of ataxia subjects (ATX) are displayed for comparison from [15]. SFW and RLW conditions are only available only for the ON state (visit #2) and the OFF state (visit #3). The different assessment points are coded with symbols. Stars denote the ON state

*Gait analysis*. Out of the pool of potentially analysable gait measures, we here adopted a hypothesis-driven approach, selecting those measures that were considered promising candidates in degenerative ataxia based on prior studies [15, 17], in particular those that had been shown to capture ataxia-related gait impairments in real-life walking behaviour [15]. These measures are primarily derived from the following gait domains: spatial and temporal step variability (*lateral step deviation* and *stride duration variability,* with higher variability values indicating worse gait performance) and gait regularity (*harmonic ratios,* with higher values indicating increased gait regularity and better gait performance). We have shown in [15] that *lateral step deviation* as well as *harmonic ratios* highly correlate with patient-reported subjective balance confidence (ABC score [18]) with the highest effect size in real-life walking behavior. In addition, both step variability measures [19] as well as harmonic ratios [20] have been shown to correlate with the risk of falls, which have a substantial impact on patients daily lives [7, 8]. For the harmonic ratios, the best sensitivity was achieved quantifying the regularity of gait in the anterior/posterior direction (*harmonic ratio*_*AP*_) [20]. We also include gait *speed* as a general (albeit unspecific) indicator of functional mobility.

At the two visits without drug (visit #1, visit #3), with a 16 month interval between them, the SARA_total_ was 11 points (visit #1, 6/21, NO drug) and 13 (visit #3, 4/23; OFF drug), respectively. Compared to the SARA score at the OFF state (visit #3, 4/23), the SARA was slightly lower (SARA_total_ = 12) in the ON state (visit #2, 3/23). However, the SARA item 'gait' showed no change between ON (visit #2) and OFF (visit #3), in both states scoring 3 points (“Considerable staggering, difficulties in half-turn, but without support”).

In contrast, the PGI-S global as well as the PGI-S domain ‘gait’ showed a change in the in the 5-point Likert scale, for both outcomes from “moderately affected” (at ON state, visit #2) to “severely affected” (at OFF state, visit #3).

Analysis of the laboratory-based walking condition (LBW) for preferred speed showed comparable values for the gait measures in the two assessments without drug (visit #1: NO; visit #3: OFF) [see Fig. 1 for *lateral step deviation* (A + B), *speed* (E + F), *harmonic ratio*_*AP*_ (I + J), and *stride duration*_CV_ (M + N)], corresponding to the slow overall progression of gait ataxia severity over this 16-month interval. In particular, for LBW slow speed, some measures showed higher values at the visit #3 compared to visit #1, e.g., lateral step deviation (Fig. 1B), which is in line with the patient’s subjective impression of more pronounced disturbances during slow walking at this later visit.

Consistent with the patient-reported impressions, gait parameters showed improvements in all ataxia-related gait measures in the ON state (visit #2) compared to the NO (visit #1) and OFF (visit #3) states—for laboratory-based walking (LBW); and also for most ataxia-related gait measures in ON (visit #2) vs OFF (visit #3)) for supervised free walking (SFW) and real-life walking (RLW) (available only for those two visits) (see Figs. 1, 2). Measures of step variability and gait regularity captured these improvements with high relative changes in ON compared to OFF (20–60%) (Fig. 2). Speed as a general (albeit unspecific) indicator of functional mobility increased also in the ON state, although relative changes were smaller compared to the—more ataxia-specific—measures step variability and regularity (Fig. 2). Notably, when comparing gait conditions, the improvements in the ON state (visit #2) in real-life walking (RLW) yielded the highest (temporal variability, speed) or second highest (spatial variability, regularity) changes compared to more constrained walking trials (LBW slow speed, LBW preferred speed, SFW) (Fig. 2).

**Fig. 2:**
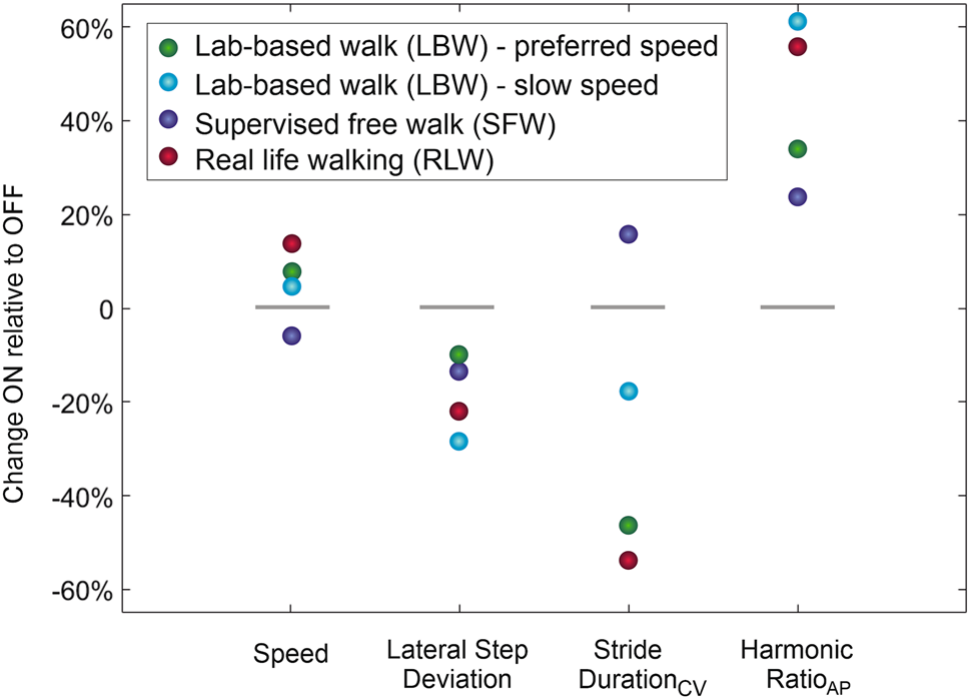
Changes in ON (visit #2) relative to OFF (visit #3) for representative gait measures in ataxia-related gait domains for different walking conditions. Changes are computed by comparison of ON (visit #2) and OFF (visit #3) gait assessments which took place just 3 weeks apart in April 2023. Changes are given for the gait domains speed, spatial variability (measure: lateral step deviation, higher variability values indicate worse gait performance), temporal variability (measure: stride duration_CV,_ higher variability values indicate worse gait performance), and regularity (harmonic ratio_AP_, higher values indicate increased regularity and better gait performance). Lateral step deviation and harmonic ratio_AP_ revealed the greatest consistency over the different walking conditions. Consistent with earlier observations [15] that found a greater influence of environmental factors on the variance of step duration in unconstrained walking, such as in the SFW condition. *CV* coefficient of variation

Treatment efficacy of 4-AP in SCA27B has so far only been demonstrated in a small number of patients and mainly on the basis of patient-reported symptom time per day and frequency of days affected by severe symptoms [2]. Objective findings unraveling the characteristics underlying these patient-reported improvements, as well as digital-motor measures which might allow to capture these effects even in everyday living and on a single-subject level, are still missing. In this structured n-of-1 assessment, we (i) provide objective digital-motor evidence that 4-AP improves ataxia-related gait characteristics in SCA27B; and that (ii) gait measures assessable by body-worn inertial sensors allow to capture and quantify 4-AP on-/off gait effects both in laboratory-based gait assessment as well as in patients’ everyday real life.

In contrast to the clinician-reported outcome widely established in ataxia clinics and treatment trials—the SARA score—which did not reflect the improvement in gait, both the patient-reported outcome as well as the digital gait outcomes in laboratory assessment and real-life walking showed specific ataxia-related improvements. In particular, gait measures in the ON state (visit #2, 3/23) were improved even compared to the drug-naïve assessment 16 months earlier (NO drug, visit #1, 6/21), indicating that the differences in gait measures between ON and OFF are not just due to a difference in (negative) motivation or other cognitive-affective factors when being in the OFF state (visit #3).

The meaningfulness of the observed changes in gait measures is supported by several lines of evidence. Previous studies have shown that higher stride variability [21] and smaller harmonic ratios are associated with higher fall risk [20]. Likewise, smaller lateral step deviation has been shown to be associated with higher patient-reported subjective confidence in real-life balance (measured by the ABC score [18]), with the largest effect consistently observed in real-life gait assessment [15]. In addition, all of these measures were assessed in our study not only in lab-based gait tasks (as in previous trials in cerebellar ataxia which assessed a decreased gait variability only in the laboratory [22, 23]), but also in patient’s real-life gait behavior. This adds further weight that the gait performance assessed here with these gait measures does not only reflect lab-based gait capability (which might potentially be of only artifact/surrogate relevance for a patient’s real life), but patient’s gait in his real life, which is key for patient relevance.

The methodological strength of our single-subject observation—as well as prior observations on the potential efficacy of 4-AP in SCA27B [2]—is the prospective intra-individual control design, with subjects serving as their own ON/OFF/NO controls. However, we cannot completely rule out a potential influence of placebo effects, although placebo effects have been observed in the previous ataxia intervention trials predominantly for (i) clinical scales (and here for appendicular rather than truncal items) and (ii) unspecific mobility measures like speed [24], and not for ataxia-related sensor gait measures specifically influenced by cerebellar-induced control impairments. However, given that all our observations are intrinsically limited by an open-label design as well the small sample size, a thorough double-blind placebo-controlled randomized-controlled trial (RCT) is required. For designing such an RCT, however, outcome measures are highly needed which—according to the recently FDA guidance on patient-focused drug developments [6]—are both objective and quantitative in nature, but at the same time also patient-relevant. Our case study indicates that gait parameters assessable by body-worn inertial sensors do indeed allow to quantify 4-AP gait effects not only in laboratory-based gait assessments, but also in—ecologically more relevant—patients’ everyday life. In fact, effect sizes might even be larger in real-life than in lab-based assessments (most probably due to increased complexity in walking behavior [25] and the larger amount of walking bouts [15]), which again presents an important information for upcoming trial design planning in SCA27B. At the same time, our findings indicate that lab-based gait assessments might here still be used to provide first surrogate snapshots of gait improvements relevant for daily life.

While warranting future validation in larger placebo-controlled cohort studies, our single-subject investigation further corroborates and objectifies the potential benefit of 4-AP on real-life gait in SCA27B. It also suggests that gait measures of step variability (in particular lateral step deviation) and gait regularity may serve as promising sensitive and meaningful and ecologically valid outcome measures, most likely resulting in significantly smaller sample size compared to the SARA score as primary outcome—as previously shown by longitudinal gait studies on SCA3 [17]. This is of immediate interest for the design of SCA27B treatment trials.

## Data Availability

The data that support the findings of this study are available on request from the corresponding author. The data are not publicly available due to privacy or ethical restrictions.
